# Supplementary material for: Aquatic Bacteria Rheinheimera tangshanensis New Ability for Mercury Pollution Removal
Source: Int J Mol Sci. 2023 Mar 5;24(5):5009. doi: 10.3390/ijms24055009 (PMC10003538; doi:10.3390/ijms24055009)
Supplement: Supplementary file 1 [file ijms-24-05009-s001.zip › ijms-2143444-supplementary.pdf]

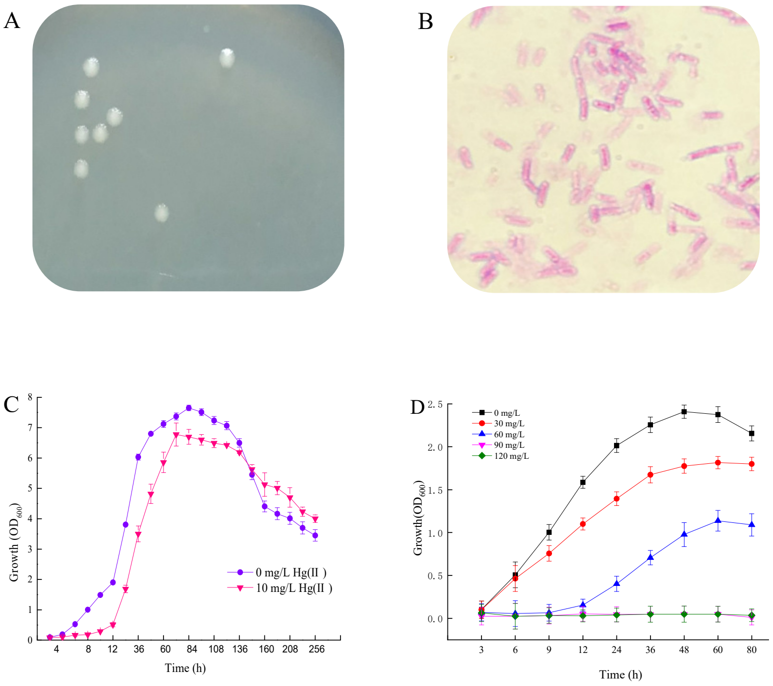

Figure S1. The character of the RTS-4 bacterial strain, including (A) colony morphology, (B) individual cell morphology after Gram staining. (C) Growth curve of RTS-4 in liquid medium containing 10 and 0 mg/L Hg(II). (D) Maximum tolerance to mercury. Bars (mean values ± SD) from three independent experiments.

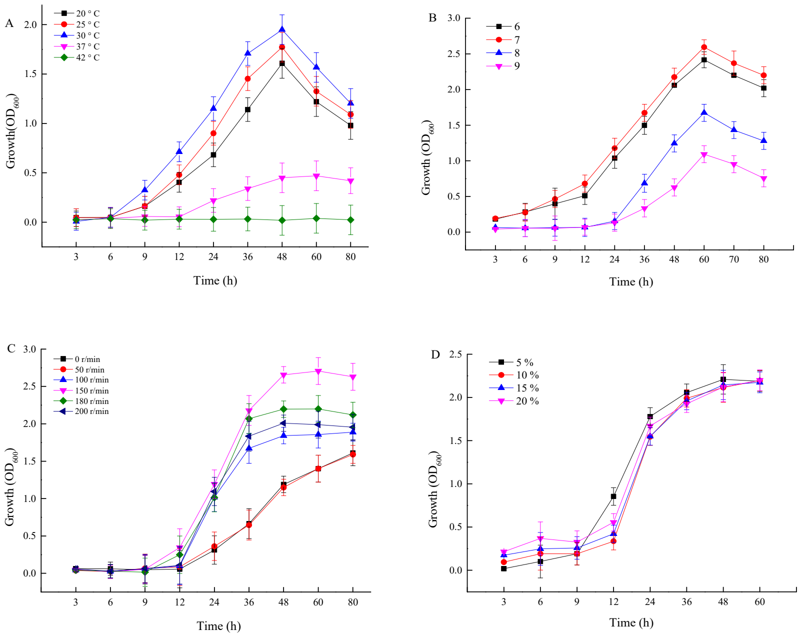

Figure S2. Optimal growth conditions of the RTS-4 bacterial strain. (A) Growth status of RTS-4 at different temperatures; (B) growth status of RTS-4 at different pH; (C) growth status of RTS-4 at different speeds; and (E) growth status of RTS-4 at different inoculation amounts. Bars (mean values ± SD) from three independent experiments.

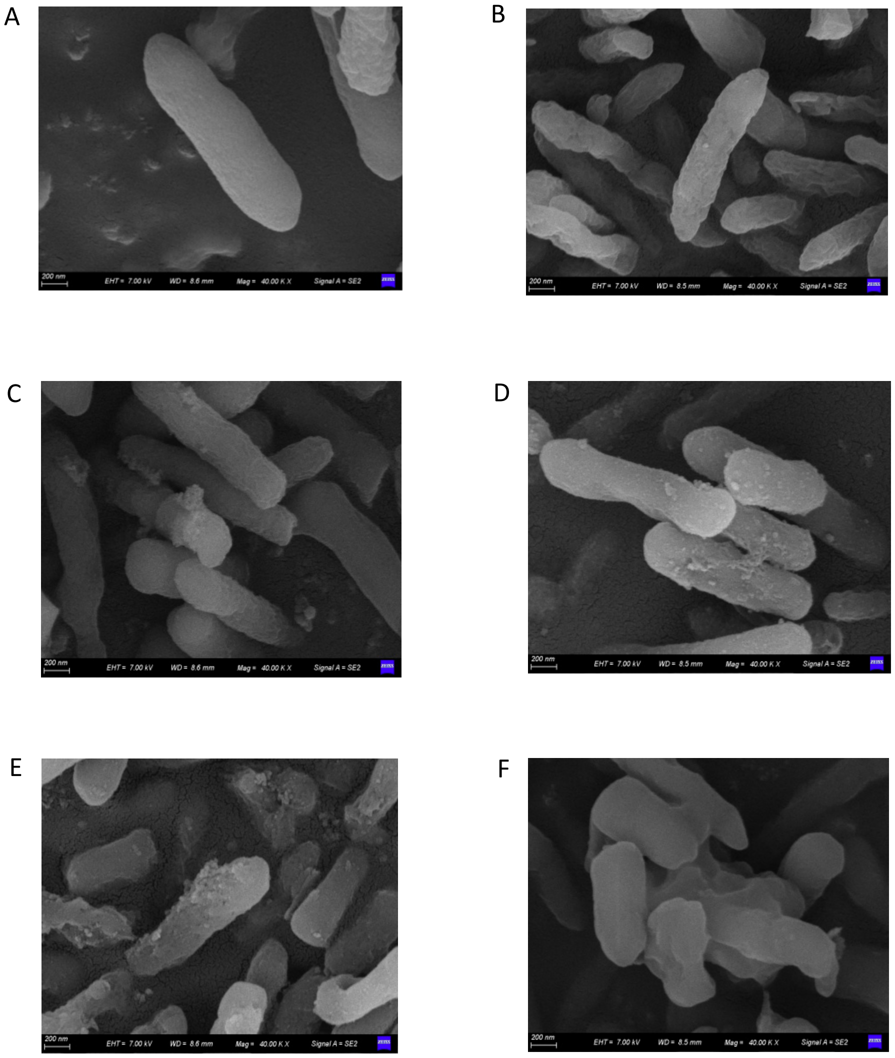

Figure S3. Scanning electron microscopy (SEM) images of the RTS-4 bacterial stains in the medium containing (A) 0, (B)10, (C) 20, (D) 30, (E) 40, and (F) 50 mg/L Hg (II). Voltage, 7.00 kV; magnification, 40.00 KX.

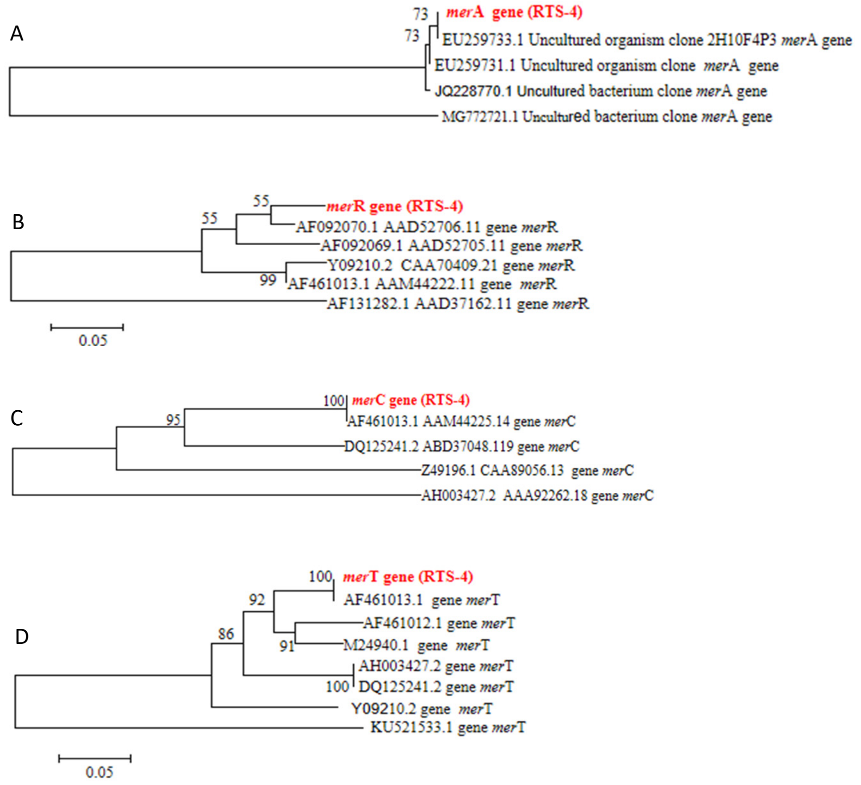

Figure S4. Gene sequence phylogenetic tree of the mer operon in RTS-4 bacteria. (A) *mer*A gene. (B) *mer*R. (C) *mer*C. (D) *mer*T. Taxonomic classifications were assessed by comparison with the NCBI Nucleic Acid Database. Numbers denote closeness distances between adjacent species. The evolutionary history was inferred using the neighbor-joining method. The bootstrap consensus tree inferred from 1000 replicates is taken to represent the evolutionary history of the taxa analyzed. Branches corresponding to partitions reproduced in less than 50% bootstrap replicates are collapsed. The percentage of replicate trees in which the associated taxa clustered together in the bootstrap test (1000 replicates) are shown next to the branches. The tree is drawn to scale, with branch lengths in the same units as those of the evolutionary distances used to infer the phylogenetic tree. The evolutionary distances were computed using the Maximum Composite Likelihood method and are in the units of the number of base substitutions per site. The analysis involved 24 nucleotide sequences. Codon positions included were 1st+2nd+3rd+Noncoding. All positions containing gaps and missing data were eliminated . Evolutionary analyses were conducted in MEGA5.

Table S1. Primers of *mer*A, *mer*R, *mer*C, *mer*T genes, and the housekeeping gene of RTS-4 bacteria.

Genes	Primers	Target fragment length
*mer*A-F	5'-GCCGTCCAAGATCATGATTC-3'	920 bp
*mer*A-R	5'-CTCGACCATCGTCAGGTAGG-3'	
*mer*R-F	5'-TATGGCGAGGCGGATGTAA-3'	406 bp
*mer*R-R	5'-CAGCAGCTCGGCTATCTCATC-3'	
*mer*C-F	5'-GCTTGGCTCAATCATCGACA-3'	469 bp
*mer*C-R	5'-CGACACCCCAACCATCAA-3'	
*mer*T-F	5'-ATGGATGAGAATCGCTCGAA-3'	425 bp
*mer*T-R	5'-GTCCTGCGCAACAAAGTAAA-3'	
16S RTS-4-F (Internal reference gene)	5'-AGGAATACCAGTGGCGAAGG-3'	1435 bp
16S RTS-4-R (Internal reference gene)	5'-CTACTCACATCACAAGGACACGAAC-3'	
